# Returning HIV‐1 viral load results to participant‐selected health facilities in national Population‐based HIV Impact Assessment (PHIA) household surveys in three sub‐Saharan African Countries, 2015 to 2016

**DOI:** 10.1002/jia2.25004

**Published:** 2017-11-24

**Authors:** Suzue Saito, Yen T Duong, Melissa Metz, Kiwon Lee, Hetal Patel, Katrina Sleeman, Julius Manjengwa, Francis M Ogollah, Webster Kasongo, Rick Mitchell, Owen Mugurungi, Frank Chimbwandira, Crispin Moyo, Vusumuzi Maliwa, Helecks Mtengo, Tepa Nkumbula, Clement B Ndongmo, Nora Skutayi Vere, Geoffrey Chipungu, Bharat S Parekh, Jessica Justman, Andrew C Voetsch

**Affiliations:** ^1^ ICAP at Columbia University New York NY USA; ^2^ Department of Epidemiology Mailman School of Public Health at Columbia University New York NY USA; ^3^ Centers for Disease Control and Prevention Atlanta GA USA; ^4^ Tropical Diseases Research Center Ndola Zambia; ^5^ Westat Rockville MD USA; ^6^ Zimbabwe Ministry of Health and Child Care Harare Zimbabwe; ^7^ Malawi Ministry of Health Lilongwe Malawi; ^8^ Zambia Ministry of Health Lusaka Zambia

**Keywords:** HIV viral load monitoring, turnaround time, TAT, return of results, population‐based surveys, PHIA

## Abstract

**Introduction:**

Logistical complexities of returning laboratory test results to participants have precluded most population‐based HIV surveys conducted in sub‐Saharan Africa from doing so. For HIV positive participants, this presents a missed opportunity for engagement into clinical care and improvement in health outcomes. The Population‐based HIV Impact Assessment (PHIA) surveys, which measure HIV incidence and the prevalence of viral load (VL) suppression in selected African countries, are returning VL results to health facilities specified by each HIV positive participant within eight weeks of collection. We describe the performance of the specimen and data management systems used to return VL results to PHIA participants in Zimbabwe, Malawi and Zambia.

**Methods:**

Consenting participants underwent home‐based counseling and HIV rapid testing as per national testing guidelines; all confirmed HIV positive participants had VL measured at a central laboratory on either the Roche CAP/CTM or Abbott m2000 platform. On a bi‐weekly basis, a dedicated data management team produced logs linking the VL test result with the participants’ contact information and preferred health facility; project staff sent test results confidentially via project drivers, national courier systems, or electronically through an adapted short message service (SMS). Participants who provided cell phone numbers received SMS or phone call alerts regarding availability of VL results.

**Results and discussion:**

From 29,634 households across the three countries, 78,090 total participants 0 to 64 years in Zimbabwe and Malawi and 0 to 59 years in Zambia underwent blood draw and HIV testing. Of the 8391 total HIV positive participants identified, 8313 (99%) had VL tests performed and 8245 (99%) of these were returned to the selected health facilities. Of the 5979 VL results returned in Zimbabwe and Zambia, 85% were returned within the eight‐week goal with a median turnaround time of 48 days (IQR: 33 to 61). In Malawi, where exact return dates were unavailable all 2266 returnable results reached the health facilities by 11 weeks.

**Conclusions:**

The first three PHIA surveys returned the vast majority of VL results to each HIV positive participant's preferred health facility within the eight‐week target. Even in the absence of national VL monitoring systems, a system to return VL results from a population‐based survey is feasible, but it requires developing laboratory and data management systems and dedicated staff. These are likely important requirements to strengthen return of results systems in routine clinical care.

## 1 Introduction

Returning results of biological laboratory testing conducted in population‐based surveys to participants offers a critical opportunity to increase engagement into clinical care to improve their health 1, 2, 3, 4, 5. Although past studies have described substantial financial, logistical and time costs associated with the return of results in the context of national surveys 1, current guidelines on population surveys that include HIV measures recommend return of HIV serostatus and viral load (VL) test results to participants 6. HIV rapid testing has made it possible to return HIV serostatus results within minutes of blood collection but it remains difficult to return VL results as testing is still mainly done at central reference laboratories.

Operationalizing the return of VL test results as part of a national population‐based survey relies on the existing infrastructure for VL testing in routine HIV clinical care. Since the release of the 2013 WHO guidelines on the use of antiretrovirals, 47 out of 52 low and middle income countries have adopted routine VL monitoring for early detection of treatment failure in their national HIV policies 7, 8. However, on‐site VL testing remains limited as documented in a recent study of 262 HIV care and treatment sites in 45 countries 9. The study found only 14% of 87 majority urban facilities in southern Africa had on‐site VL testing capabilities in 2014 9.

In this context of limited and concentrated capacity of VL testing in urban centers, ensuring that VL test results are returned in a timely manner to survey participants in a large, national population‐based survey requires development of survey‐specific specimen and data management systems. In order to obtain quality VL results, specimens must be well collected, transported under proper conditions, and monitored for arm‐to‐freezer time from participants’ homes to specialized laboratories for processing and testing. Adding to the complexity, a mechanism to interface with existing VL testing instruments and laboratory information systems (LIS) at the laboratories must be established to handle survey specimens apart from clinical specimens. Return of VL results in the clinical setting has been described in past studies 10, 11, 12, 13. To date, there is a dearth of data on process and system adaptations needed to return VL results with acceptable turnaround time (TAT) in the context of population based surveys identifying ~2500 HIV positive individuals spread nationwide 14. In a small population‐based household survey in two rural sub‐districts in South Africa, Lippman, et al. used a study phone number for 158 HIV positive participants to call to obtain VL results but did not report TAT 14.

The Population‐based HIV Impact Assessment (PHIA) Project, implemented by ICAP at Columbia University in collaboration with the Ministries of Health, US Centers for Disease Control and Prevention (CDC) and other partners, is assessing the status of the HIV epidemic in 13 countries in sub‐Saharan Africa (SSA) and Haiti by measuring nationally representative, population‐level HIV prevalence, incidence and VL suppression. In the context of national household surveys, the PHIA project established a system to measure VL among HIV+ individuals and return results to their preferred health facility within eight weeks of the sample collection. Herein, the processes and systems established to return VL results and the TAT achieved in the first three surveys in Zimbabwe (ZIMPHIA), Malawi (MPHIA), and Zambia (ZAMPHIA) are described.

## 2 Methods

Consenting participants aged 0 to 64 years in ZIMPHIA and MPHIA and 0 to 59 years in ZAMPHIA underwent in‐home counseling and HIV rapid testing according to each country's HIV national testing guidelines 15, 16, 17. The consent forms specifically referred to blood draw, home‐based HIV rapid testing and, depending on the country, other point of care tests, such as syphilis, hepatitis B surface antigen (HBsAg), and CD4. The consent forms also referred to receiving at a nearby healthcare facility VL test results that came from assays conducted at central laboratories. Depending on age, whole blood was collected from participants either by venous blood draw, finger prick or heel stick for household‐based testing and additional biomarker testing at the laboratory. At the end of each collection day, all field specimens were shipped to pre‐selected Ministry of Health (MOH) district laboratories with project‐specific lab capacity enhancements near the area of field work for processing into plasma and/or dried blood spot (DBS) specimens, quality assurance (QA) testing and −20°C freezer storage. Approximately weekly, the plasma and DBS specimens were then shipped to a central reference laboratory to conduct specialized tests, including VL testing on HIV+ specimens and long‐term storage at −70°C or below.

### 2.1 Viral load testing

Samples from all HIV positive participants were tested for VL levels using an automated platform at a central reference laboratory in each country. For ZIMPHIA and ZAMPHIA, the COBAS^®^ AmpliPrep/COBAS^®^ TaqMan^®^ HIV‐1 Test, v2.0 was performed on the Roche (Pleasanton, CA) platform while the Abbott m2000rt System (Chicago, IL, USA) using the Abbott Real Time HIV‐1 Assay was performed in MPHIA, both according to the manufacturers’ instructions. The primary specimen type for VL testing was plasma, but in 4 to 5% of all cases and <1.1% of positive cases only DBS samples were available; DBS were tested using a modified Abbott assay for DBS elution in MPHIA and ZAMPHIA, while the NucliSENS easyQ^®^ (bioMérieux, Marcy‐l’Étoile, France) platform was used for DBS VL testing in ZIMPHIA. On a weekly basis, trained laboratory scientists on the PHIA Project reviewed viral load results for quality control and quality assurance.

### 2.2 Data management

**Figure 1:**
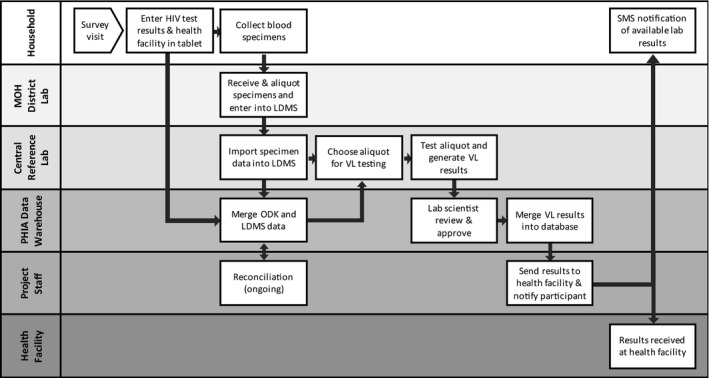
Return of viral load results data flow for the PHIA Project. Each row represents different domains data travel starting from the household, MOH district laboratories, central reference laboratory, the PHIA data warehouse and project staff at the ICAP office or the central reference laboratory. MOH, Ministry of Health; ODK, open data kit questionnaire software application; LDMS, laboratory data management system.

For the entire PHIA Project, a centralized data management system was established to process and monitor return of results (RoR) across each country (Figure 1). Extensive preventative steps were taken to ensure that all personally identifiable data were securely stored with limited access to a few select individuals to ensure participant confidentiality. In the home, along with blood collection, the participant's age, unique participant identification number (PTID), HIV rapid test results and preferred health facility to collect the VL results were captured in an Open Data Kit (ODK) software application configured and implemented on Google Nexus 9 Android tablets. Blood tubes collected in the household were affixed with PTID labels, and received and logged according to date and time at the MOH district laboratories using a laboratory data management system (LDMS, Frontier Science, Boston, MA). LDMS generated labels with unique specimen IDs for each plasma and DBS aliquot derived from an individual participant blood sample. Plasma and DBS storage time and location in individual freezers were also recorded in LDMS. LDMS data files and shipment reports were generated each week and sent with plasma and DBS specimens to the central reference laboratory where the specimen data were imported into the LDMS.

A data sharing architecture was developed to securely transmit the data at least weekly from the field teams and central reference laboratories to a data warehouse hosted on a central PHIA server (Figure 1). Twice weekly, participant data from ODK and specimen data from LDMS were merged using the PTIDs in the central data warehouse. For records that successfully merged, laboratory orders for VL testing, containing PTID, unique specimen ID, and specimen location were generated using a program written in SAS for Windows version 9.3 (SAS Institute Inc., Cary, NC, USA). For MPHIA and ZAMPHIA, orders were posted to a secure file transfer protocol (FTP) server for lab technicians to download; for ZIMPHIA orders were submitted via an XML (extensible markup language) interface directly into the laboratory information system (LIS) at the central reference laboratory. Based on the VL testing orders, technicians retrieved the specimens and performed the VL testing. For records that were not successfully merged, reconciliation efforts were made by contacting field teams and MOH district lab teams to retrieve paper documentation to verify and correct erroneous entries into ODK or LDMS.

For ZIMPHIA and MPHIA, the VL test instruments were connected to the existing local LIS and individual participant results were generated and printed in report form from the LIS in the same manner as routine clinical testing. For ZAMPHIA, a laboratory data manager exported instrument files in CSV (comma separated values) format and uploaded on a bi‐weekly basis to the central data warehouse, where a SAS program was run to generate participant result reports as PDF (portable document file) files fashioned after laboratory results reports used for clinical patients. The PDF files were then downloaded and printed at the central reference laboratory and mailed out using the national courier system. For participants who had chosen a facility with an active Project Mwana account, an SMS system managed by the Zambia Ministry of Health to deliver HIV‐related test results to health facilities, a CSV file containing results, facility, and location information was created and fed into the system to send results to the health facility and reminders to participants (https://www.rapidsms.org/projects/project-mwana/) 18.

### 2.3 Reporting VL results to health facilities

In addition to laboratory orders, twice a week a contact list of HIV+ participants identified by PHIA surveys was generated using a SAS script running off the central data warehouse. It contained each participant's PTID, unique specimen ID, data collection date, field HIV test result, sample type (plasma/DBS), first name, last name, address, phone number (optional), and preferred health facility name. The project staff based in the ICAP office (ZIMPHIA/MPHIA) or the central reference laboratory (ZAMPHIA) downloaded this list to track the status of the VL result report for each HIV+ participant. For ZIMPHIA and MPHIA, the administrative officer and the information, communication and technology (ICT) officer worked to coordinate the RoR process. In ZAMPHIA, a dedicated RoR coordinator was recruited and worked directly with the laboratory team performing the VL testing. In ZIMPHIA and MPHIA, the participant VL result reports were sent via the national courier system or project driver. In ZAMPHIA, in addition to the national courier system, results were sent through a new module created in Project Mwana to transmit viral load results. During the household visit, all participants were told to visit the health facility to collect the test results after eight weeks. Participants who provided cell phone numbers received text messages regarding availability of test results from the project staff or Project Mwana. Project staff verified identification of the recipient using security questions provided during the interview prior to sending the messages. For a small minority of results with missing or invalid health facility information, VL results were sent to health facilities selected by the other participating household members or all health facilities in the catchment area of the participant's home.

### 2.4 Monitoring and analysis

Monthly summary statistics were generated to track each stage of the RoR process from specimen processing and merging of participant interview, specimen, and VL results data, to generation and couriering of participant results reports to health facilities. Median time from blood draw to results received at the health facility was also tracked, disaggregated by month, residence (urban vs. rural), and RoR stages. The ROR stages were divided into 3 stages: stage 1 was time from blood draw to receipt at central reference laboratory; stage 2 was from receipt at central reference laboratory to the availability of approved VL test result; and stage 3 was time from availability of approved VL test result to results delivered to the health facility. We used Wilcoxson Rank Sum tests to compare median TAT by facility characteristics.

### 2.5 Ethics statement

Survey protocols for the Zimbabwe, Malawi and Zambia PHIAs were approved by the Centers for Disease Control and Prevention Institutional Review Board (IRB), the Columbia University Medical Center IRB, and relevant local regulatory bodies, including the Medical Research Council of Zimbabwe, National Health Science Research Committee of Malawi, and the Tropical Disease Research Center in Zambia.

## 3 Results and discussion

### 3.1 Returning VL results

**Figure 2:**
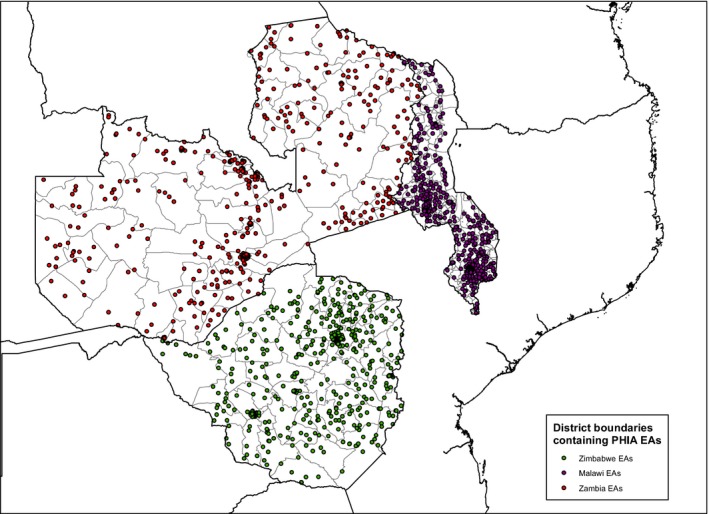
PHIA enumeration areas across the 196 districts in ZIMPHIA, MPHIA, and ZAMPHIA, 2015 to 2016.

In ZIMPHIA, MPHIA, and ZAMPHIA, a total of 78,090 participants provided a blood sample from 29,634 households located in 196 districts combined (Table 1; Figure 2). The three surveys identified 8391 HIV+ participants, of which, 8313 (99.1%) were tested for VL levels. Of the VL tests successfully run, 8245 (99.2%) were returned to the health facility. There were 68 results that were unable to be delivered to the specific health facilities selected by participants due to missing participant contact or health facility information (n = 43), unresolved PTID entry errors (n = 11), discrepant serology results that required household revisits after survey completion (n = 13), and instrument failure (n = 1). Per protocol, these results were sent to health facilities selected by other consenting household members or health facilities in the catchment area of the participant's home.

**Table 1:** Participants interviewed and tested for HIV in ZIMPHIA, MPHIA, and ZAMPHIA, 2015 to 2016

	Total	ZIMPHIA	MPHIA	ZAMPHIA
Total participants	78,090	27,609	23,353	27,128
Adults	56,877	20,577^a^	17,187^a^	19,113^b^
Children (0 to 14)	21,213	7032	6166	8015
Households with participants	29,634	10,897	9359	9378
Number of districts	196	91	31	74

a 15 to 64 year.

b 15 to 59 year.

### 3.2 TAT for sending VL results to health facilities

In ZIMPHIA and ZAMPHIA where return dates were systematically recorded, 5082 of 5979 (85.0%) VL results were returned within eight weeks of blood draw (Table 2). Return dates were unavailable in approximately 5% (ZIMPHIA) to 10% (ZAMPHIA) of cases and, in those instances, dates sent by project staff were used. The exact timing of return of results in MPHIA is unavailable as dates VL results were delivered to health facilities were not systematically captured; however based on dates when RoR logs were shared from project staff in country with the central data management team, all results were returned within 75 days. Median time from blood draw to results being sent back to the health facility in ZIMPHIA and ZAMPHIA was 48 days (IQR range: 33 to 61 days) (Table 3) with similar results for participants living in urban and rural areas (46 days vs. 49 days, respectively; *p* = 0.35) (Table 3). TAT was significantly longer for specimens collected in the first month of survey implementation and shortened after the second month of each study (55 days vs. 44 days, respectively; *p* < 0.01). Overall, stage 3 of the RoR process (time from approved VL test results to delivery of results to the health facility) took the longest, with a median of 26 days (IQR range: 16 to 43 days). In Zimbabwe, stage 3 required a median of 36 days, while in Zambia, only 21 days was needed to complete this stage. Stage 1 (time from blood draw to receipt of specimens at the central reference laboratory) was the shortest interval at 7 to 8 days in all countries. There was substantial variation by country in time required for stage 2: while in Zimbabwe and Zambia it took medians of 9 and 11 days respectively, in Malawi, it took 20 days to provide VL results after receiving specimens at the laboratory.

**Table 2:** Summary Statistics for return of VL results in ZIMPHIA, MPHIA, and ZAMPHIA, 2015 to 2016

	Total	ZIMPHIA	MPHIA	ZAMPHIA
	n (%)	n (%)	n (%)	n (%)
HIV+ participants identified	8391	3505	2326	2560
VL test performed	8313 (99.1)	3499 (99.8)	2315 (99.5)	2499 (97.6)
Returned	8245 (99.2)	3481 (99.5)	2266 (97.9)	2498 (99.96)
Within eight weeks	5082 (85.0)^a^	3104 (89.2)	N/A	1978 (79.2)
After eight weeks	773 (13.1)^a^	360 (10.3)	N/A	413 (16.5)
Unknown sent date	124 (2.0)^a^	17 (0.5)	N/A	107 (4.3)
Not returned	68 (0.8)	18 (0.5)	49 (2.1)	1 (0.04)
VL test not performed	78 (0.9)	6 (0.2)	11 (0.5)	61 (2.4)

a Includes only ZIMPHIA and ZAMPHIA results. Denominator is 5979 VL test returned in ZIMPHIA and ZAMPHIA.

**Table 3:** Median TAT (IQR; range) in days for return of results in ZIMPHIA, MPHIA and ZAMPHIA, 2015 to 2016

	Total (days, IQR)	ZIMPHIA (days, IQR)	MPHIA (days, IQR)	ZAMPHIA (days, IQR)
Median number of days from blood draw to health facility	48 (33 to 61)	52 (36 to 62)	N/A	41 (31 to 57)
Returned to urban participants	46 (34 to 62)	53 (37 to 64)	N/A	42 (31 to 57)
Returned to rural participants	49 (33 to 60)	51 (35 to 60)	N/A	40 (30 to 57)
Month 1	55 (43 to 70)	55 (45 to 63)	N/A	55 (42 to 84)
Month ≥2	44 (32 to 58)	51 (35 to 61)	N/A	36 (27 to 46)
Stage 1: Blood draw to receipt at central reference laboratory	8 (5 to 10)	8 (5 to 10)	7 (5 to 11)	8 (5 to 11)
Stage 2: Receipt at central reference laboratory to approved VL test results	12 (7 to 23)	9 (6 to 14)	20 (12 to 27)	11 (7 to 20)
Stage 3: Approved VL test result to delivery of results to health facility	26 (16 to 43)^a^	36 (20 to 50)	b	21 (14 to 29)

a Includes only ZIMPHIA and ZAMPHIA results.

b In Malawi, exact dates are unavailable but returnable results reached health facilities by 11 weeks or 75 days from blood draw based on dates when RoR logs were shared with central data management team.

## 4 Discussion

To our knowledge this study is the first to describe the RoR processes and systems for concurrent, national population‐based HIV surveys in SSA. In the PHIA surveys in Zimbabwe, Malawi and Zambia, we achieved high rates of success in VL testing along with high quality questionnaire data that allowed for the vast majority of HIV+ participants receiving their VL results at their health facility of choice within eight week of blood collection. Furthermore, despite the additional complexities resulting from venous blood collection, transportation of specimens from participants’ homes, and use of survey‐specific systems to order VL tests and prepare and deliver participant VL results reports, the median time from blood draw to VL results being returned to the health facility of 48 days was on par with the return of clinical VL results documented in past studies 10, 11, 12, 13. For example, a recent assessment of national VL monitoring scale up in seven sub‐Saharan African (SSA) countries found that TAT ranged from three and four days in South Africa and Namibia to 42 and 50 days in Malawi and Cote d'Ivoire 10. Careful planning and intensive training of field and laboratory staff to ensure timely transportation and management of specimens as well as on‐going monitoring of each positive specimen from blood draw to VL results being sent to health facilities were critical in achieving the TAT goals in the PHIA project.

The first set of PHIA surveys provided a critical lesson on the importance of recruiting dedicated RoR staff that can work directly with laboratory staff at the central reference laboratory to implement RoR processes. The median TAT decreased from the second month onward in ZAMPHIA which coincided with the recruitment of a dedicated RoR coordinator. In ZIMPHIA and MPHIA where the RoR activities were managed by staff with other core responsibilities, TAT either did not significantly improve (ZIMPHIA) or adequate documentation on return dates was not maintained (MPHIA). The RoR coordinator served a critical role of tracking, for each HIV+ participant identified in ZAMPHIA, the specimen location, contact information and VL test result originating from different data sources. In addition, the RoR coordinator kept track of elapsed time since the date of blood collection to ensure processes and systems ran smoothly to meet the eight week goal.

In all three countries, we found that the existing RoR systems for clinical care were only partially adaptable for use for the PHIA surveys. To be able to track and confirm the status of each VL result from test order to receipt at health facility in a timely manner required the creation of a robust data system that allowed staff on a weekly basis to monitor the progress and identify bottlenecks. The development of the system required highly skilled programmers and data managers. However, the system has proven to be replicable within the context of the PHIA Project and is being implemented with minimal adaptations in subsequent PHIA surveys. Investing in a national integrated RoR system that accommodates both clinical and surveillance results will be important as countries expand viral load monitoring capacity. In addition, use of point of care VL test instruments, including Alere™ q HIV‐1/2 Detect assay and Cepheid Xpert HIV‐1 VL assay, may offer advantages in future surveys to minimize TAT and avoid the complexities arising from specimen transport, handling and data management issues described above 19. However, these instruments are best suited for decentralized health facilities that are remote, allowing for rural HIV positive patients without access to central laboratories to be clinically monitored in their disease progression. They may not be suited for national surveillance programs with limited budgets and requirements for high‐throughput platforms.

The high level of RoR in the first three PHIA surveys is particularly encouraging as return of clinically relevant laboratory test results, such as VL levels for HIV+ individuals, could serve as a useful strategy to incentivize participation by those who already know their HIV+ status and are engaged in clinical care. With expanded access to HIV testing and treatment services in the past decade in SSA, some population‐based surveys have found decreased participation by this group, resulting in underestimation of HIV prevalence 20, 21. Given the importance of population‐based HIV surveys to monitor the population‐level progress toward the global “90‐90‐90” HIV treatment targets, employing strategies to incentivize participation by such groups is critical 6.

Our RoR had several limitations. In Malawi, because we did not recruit an RoR coordinator, it was challenging for project staff with competing priorities to keep track of delivery dates on a consistent basis. Additionally, the long distance between the project office and central reference laboratory added complexity to communications and coordination of RoR, evidenced by the longer interval experienced from approved VL results to delivery of results to health facility (stage 3), compared to Zimbabwe and Zambia. A small minority of delivery dates to the facility recorded for ZIMPHIA and ZAMPHIA were dates project staff sent out the results, such as when the national courier systems were used,, while when project vehicles or Project Mwana were used delivery dates were tracked by the driver or the Project Mwana system. The TAT summarized here may therefore be a slight underestimate.

The first three surveys did not collect any data on whether potential participants decided to take part in the survey because of the offer to learn about their VL results. There are no data on what happened after the VL results arrived at the health care facility, therefore there are no data on how many participants learned their VL results or whether the VL results were used by clinicians to evaluate treatment progress. Future PHIA surveys will track these additional data at a minimum in a sample of health facilities to better understand how survey participants use the PHIA results to improve their health. However, confirming receipt of VL results for all participants requires visiting hundreds of health facilities nationwide and retrieving participant records from existing clinic data systems after the conclusion of the survey. Substantial human and logistical resources are needed that may not be feasible for many HIV surveillance projects.

## 5 Conclusions

The PHIA Project surveys in Zimbabwe, Malawi and Zambia have demonstrated that returning VL results in the context of a national population‐based survey is feasible, but requires establishing specimen and data management systems to allow for tracking each participant result to ensure timely return as specified in the survey protocols. Having dedicated data management staff and RoR coordinators facilitated timely return of VL results. These are likely important requirements to strengthen RoR systems in routine clinical care.

## 6 Competing interests

The authors have no competing interests to declare.

## 7 Authors’ contributions

SS, YTD, MM, JEJ, and ACV conceptualized and drafted the manuscript. SS, MM, and KL conducted the data analysis. HP, KS, JM, FMO, WK, RM, OM, FC, CM, VM, HM, TN, NSV, GC, CN, BP, JEJ, and ACV provided critical inputs in the draft manuscript.
